# Area Postrema Syndrome: A Rare Feature of Chronic Lymphocytic Inflammation With Pontine Perivascular Enhancement Responsive to Steroids

**DOI:** 10.3389/fneur.2020.00730

**Published:** 2020-08-18

**Authors:** Weihe Zhang, Lei Cui, Mingrui Dong, Zhaohui Tian, Yujuan Jiao, Jinsong Jiao

**Affiliations:** Department of Neurology, China-Japan Friendship Hospital, Beijing, China

**Keywords:** CLIPPERS, neuroinflammation, neuromyelitis optica spectrum disorders, MRI, area postrema, misdiagnosis

## Abstract

**Background:** The area postrema syndrome (APS) is a unique diagnostic criterion for neuromyelitis optica spectrum disorders (NMOSD). However, APS has rarely been reported in cases of chronic lymphocytic inflammation with pontine perivascular enhancement responsive to steroids (CLIPPERS).

**Case presentation:** A 36-year-old woman presented with APS and clinical features of diffuse central nervous system involvement during the early stage of the disease. Owing to the absence of serum aquaporin 4 antibodies, she was initially misdiagnosed as a case of seronegative NMOSD. However, the distinct neuroimaging characteristics [symmetrical small punctuate gadolinium enhancing lesions (pepper-like)], typical clinical/radiological relapse, and intense steroid-dependence in this case, prompted us to correct the diagnosis as probable CLIPPERS. To prevent relapse, long-term oral steroids and an immunosuppressive agent were administered.

**Conclusions:** CLIPPERS may present as APS, and should be considered in the differential diagnosis of NMOSD.

## Background

Chronic lymphocytic inflammation with pontine perivascular enhancement responsive to steroids (CLIPPERS) is a rare chronic central nervous system (CNS) inflammatory disorder of unknown origin. The condition was first described by Pittock et al. (1). CLIPPERS is characterized by distinct radiologic features on magnetic resonance imaging (MRI), i.e., punctate and curvilinear enhancing lesions in pons and cerebellum (1, 2). However, other well-characterized diseases such as CNS lymphoma and some autoimmune diseases may mimic the clinical and radiological features of CLIPPERS. In addition, a definitive diagnosis of CLIPPERS is typically challenging owing to the absence of specific biomarkers and the lack of availability of pathological materials for most patients (3). In this study, we report on a patient who presented with intractable vomiting and hiccups (IVH) and area postrema (AP) lesion, but without serum aquaporin 4 antibodies (AQP4-IgG). To the best of our knowledge, these features have never been reported in a patient with CLIPPERS. The presence of AP syndrome (APS) in this patient made the diagnosis challenging and highlights the importance of differential diagnosis in such cases.

## Case Presentation

A 36-year-old woman was admitted to the China-Japan Friendship Hospital (Beijing, China) in January 2019 with chief complaints of constipation since 9 months, facial numbness and hearing loss in the right ear since 3 months, intractable vomiting and hiccups, dizziness, and unsteady gait since 1 month, and progressive weakness since 15 days. Prior to admission, she was administered some traditional Chinese medicines and symptomatic therapies at primary hospitals; however, she did not respond to treatment. She had no remarkable past medical history. On admission, she exhibited malaise and was not able to walk; however, she was conscious and well-oriented to time, place and person. She had diplopia and right-ear hearing loss. Muscle strength in the bilateral extremities was graded as 3/5 (Medical Research Council). There were signs of bilateral tendon hyperreflexia and positive Babinski. Bilateral finger-nose and heel-knee-tibia tests were abnormal. In addition, she had a sensory level at the 7th thoracic segment. The modified Rankin scale (mRS) score at presentation was 4.

**Figure 1:**
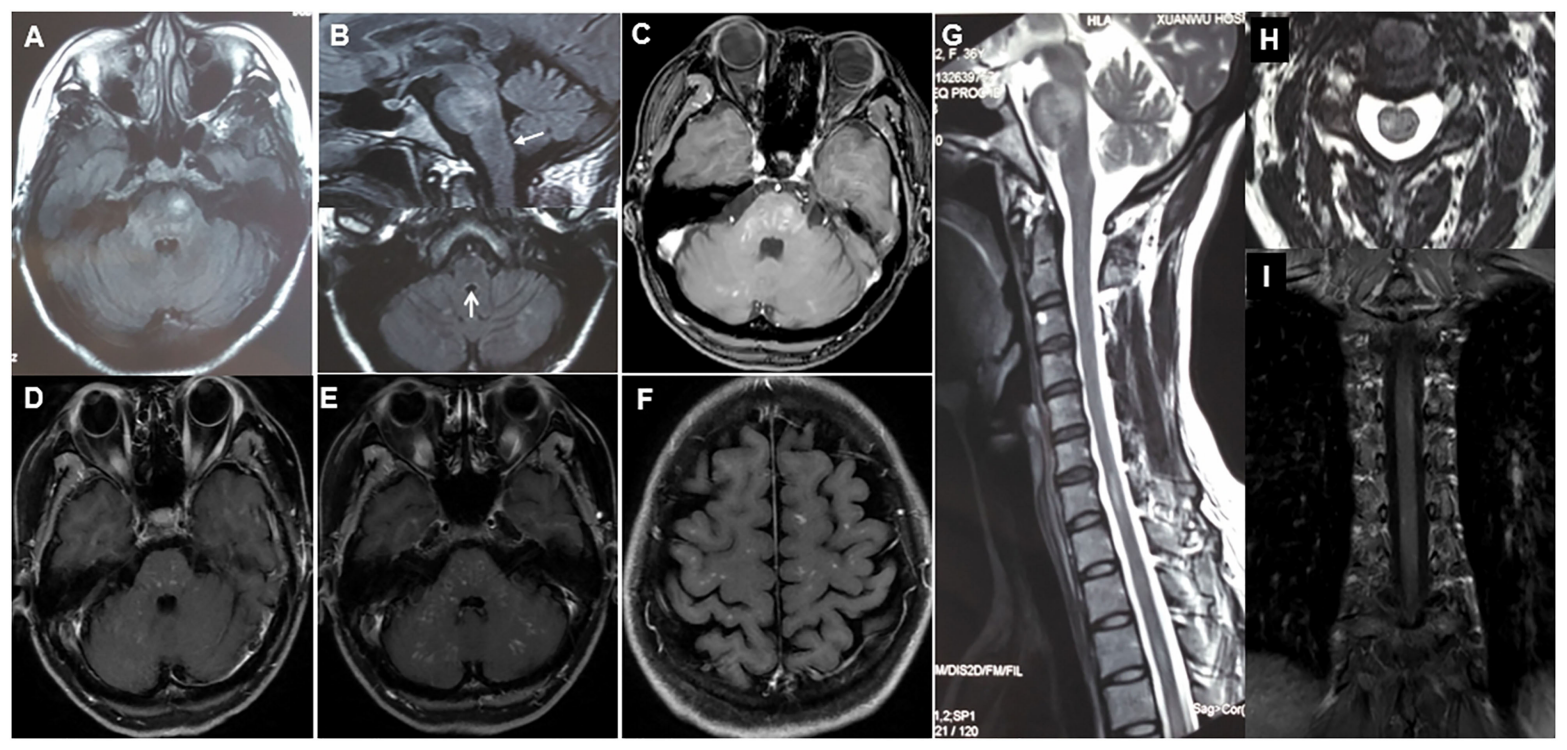
**(A–C)** Initial cerebral MRI (axial T2 FLAIR) and corresponding T1 post-gadolinium image showing disseminated symmetrical contrast-enhanced lesions in bilateral pons, cerebellum, and area postrema (arrows). **(D)** Three months after the initial steroid therapy, axial T1 post-gadolinium enhanced brain MRI shows a decrease in the extent of abnormal gadolinium enhancement. **(E,F,I)** At relapse, axial T1 post-gadolinium brain MRI and coronal spinal MRI shows symmetrical punctate and curvilinear enhancing lesions in bilateral brainstem, cerebellum, periventricular and subcortical white matter, and spinal cord. **(G,H)** Sagittal and axial spine MRI shows multiple small lesions in cervical and thoracic spinal segments.

Examination of cerebrospinal fluid (CSF) revealed normal cell count and a mild elevation of protein concentration [0.47 g/L (reference range, 0.15–0.45 g/L)]. Magnetic resonance imaging (MRI) of the brain (Dec. 2018) exhibited disseminated symmetrical lesions distributed in the bilateral pons, cerebellum, AP, and periventricular and subcortical white matter; the lesions showed gadolinium enhancement (Figures 1A–C). Spine MRI exhibited multiple lesions in cervical and thoracic spinal segments (Figures 1G,H). Whole body computed tomography revealed no abnormalities. Furthermore, no abnormalities were detected on oligoclonal bands (OCB), AQP4-IgG [ELISA by EUROIMMUN (China) Co., Beijing, China], myelin oligodendrocyte glycoprotein antibodies [MOG-IgG, indirect immunofluorescence assay by EUROIMMUN (China) Co., Beijing, China], glial fibrillary acidic protein antibodies [GFAP-IgG, cell-based assay by EUROIMMUN (China) Co., Beijing, China], ganglioside antibodies in serum and CSF, or antibodies associated with autoimmune encephalitis and paraneoplastic syndrome. Metagenomic next-generation sequencing of CSF for microbial detection was also normal.

The patient was initially diagnosed as having seronegative NMOSD and was prescribed intravenous methylprednisolone (1,000 mg per day for 5 days) followed by tapered oral prednisone (60 mg per day). One month later (Feb. 2019), the patient showed marked alleviation of clinical symptoms and regained her walking capability (mRS score 1). At the 3-month follow-up (April 2019), she had fully recovered with distinctly reduced abnormal gadolinium enhancement on cranial contrast MRI (Figure 1D).

However, 5 months (Jun. 2019) after the initial immunosuppressive therapy, the patient experienced a relapse with dizziness, diplopia, and unsteady gait; at that time the dose of oral prednisolone was decreased to 30 and 5 mg, every other day. Repeat contrast MRI showed recurrence of diffuse, symmetrical small punctate gadolinium enhancing lesions (pepper-like) in the brain (especially pons) and spinal cord (Figures 1E,F,I). Repeat examination of serum AQP4-IgG showed negative results. Therefore, she was reassessed and diagnosed with CLIPPERS and was administered intravenous methylprednisolone (500 mg per day for 5 days). One month (Jul. 2019) later, her symptoms were remarkably alleviated. To prevent further relapse, along with a tapered regimen of oral prednisone (much slower than the first time), she was subsequently prescribed tablet mycophenolate mofetil (750 mg, twice daily). Three months (Sep. 2019) after the last relapse, she was able to walk independently and only complained of mild facial numbness.

## Discussion

The features in our patient that stood out the most were the IVH symptoms caused by AP involvement, that lasted for 4 weeks at the disease onset. AP is the most important circumventricular organ. It is an emetic reflex center that is not protected by the blood–brain barrier; therefore, it is believed to be the first organ to be attacked by AQP4-IgG in patients with NMOSD. APS is characterized by clinical symptoms of IVH in the context of a lesion in the AP. APS was included as a core clinical criterion of NMOSD (4) and is a typical heralding syndrome in patients with NMOSD (5, 6). The clinical features of MOG-IgG associated disorders (MOGAD) often overlap with those of AQP4-IgG seropositive NMOSD, such as optic neuritis and transverse myelitis. AP lesions and IVH can unexpectedly occur in a few MOGAD patients (7); however, this is a rare association because MOG is expressed throughout the CNS and is not specific to the AP area. Therefore, our patient was initially presumptively diagnosed as a case of seronegative NMOSD considering the absence of AQP4-IgG and MOG-IgG.

However, after the patient's relapse, the following conditions caught our attention: progressive disease course in the early stage, lack of typical imaging findings of NMSOD, persistence of gadolinium enhancement >3 months following steroid therapy, repeated seronegative AQP4-IgG, and typical clinical/radiological relapse. Since these conditions are not commonly encountered in NMOSD (4, 8), it prompted us to reassess the previous diagnosis. Our patient exhibited the core clinical and imaging features of pontocerebellar dysfunction compatible with pepper-like gadolinium enhancing lesions which were responsive to corticosteroid therapy. Since these features conformed to the criteria proposed by the Mayo Clinic (2), we corrected the diagnosis as probable CLIPPERS. Due to the rapid resolution of cerebellar lesions in our patient, a histopathologic analysis was not performed to arrive at a definitive diagnosis. It is worth mentioning that a brain biopsy is not mandatory in the presence of typical features of CLIPPERS (1).

In addition, extra-brainstem-cerebellar (cerebral white matter and spinal cord) gadolinium enhancing lesions are found in more than half of all patients with CLIPPERS (9). Consistently, these atypical findings in our patient distracted us. First of all, the absence of OCB in CSF and steroid over-dependence made the diagnosis of multiple sclerosis unlikely. Second, although our patient exhibited hearing loss, the diagnosis of Susac syndrome was excluded owing to the lack of encephalopathy and central corpus callosal lesions on the T2-weighted sequence; in addition, spinal cord involvement is extremely rare in Susac syndrome (10). The likelihood of anti-GFAP encephalomyelitis was also minimal given that the meninges were spared and our patient had a negative GFAP-IgG status (11). Other diagnoses including vasculitic, infectious, lymphomatous, and granulomatous etiologies were also eliminated through extensive tests. Moreover, due to the vulnerable anatomical location, it is not surprising to have vertigo and vomiting as presenting features in patients with CLIPPERS (1, 2, 9). However, bulbar involvement and intractable hiccups are rare in CLIPPERS (9); to the best of our knowledge, the typical presentation of APS (as seen in the present case) has never been reported in a case of CLIPPERS.

In summary, the current case introduces APS as a novel clinical feature of CLIPPERS. This case report also highlights the need to include CLIPPERS in the differential diagnosis of NMOSD. These results may help broaden the clinical-radiological spectrum of CLIPPERS.

## Data Availability Statement

All datasets generated for this study are included in the article/supplementary material.

## Ethics Statement

The studies involving human participants were reviewed and approved by Institutional Review Board of China-Japan Friendship Hospital. The patients/participants provided their written informed consent to participate in this study. Written informed consent was obtained from the individual(s) for the publication of any potentially identifiable images or data included in this article.

## Author Contributions

WZ drafted the manuscript. WZ, LC, MD, ZT, and JJ prepared the materials, collected and analyzed the data. WZ and JJ revised the manuscript. All authors contributed to the article and approved the submitted version.

## Conflict of Interest

The authors declare that the research was conducted in the absence of any commercial or financial relationships that could be construed as a potential conflict of interest.

## Abbreviations

APS: area postrema syndrome
AQP4-IgG: aquaporin 4 antibodies
CSF: cerebrospinal fluid
IVH: intractable vomiting and hiccups
MOG: myelin oligodendrocyte glycoprotein
GFAP: glial fibrillary acidic protein
mRS: modified Rankin scale
OCB: oligoclonal bands.
